# GATA4 regulates angiogenesis and persistence of inflammation in rheumatoid arthritis

**DOI:** 10.1038/s41419-018-0570-5

**Published:** 2018-05-02

**Authors:** Wanwan Jia, Weijun Wu, Di Yang, Chenxi Xiao, Mengwei Huang, Fen Long, Zhenghua Su, Ming Qin, Xinhua Liu, Yi Zhun Zhu

**Affiliations:** 1 Shanghai Key Laboratory of Bioactive Small Molecules, Department of Pharmacology, School of Pharmacy, Fudan University, Shanghai, 201203 China; 2 State Key Laboratory of Quality Research in Chinese Medicine and School of Pharmacy, Macau University of Science and Technology, Macau, China

## Abstract

Rheumatoid arthritis (RA) is a chronic autoimmune disease characterized by abnormal inflammation, angiogenesis, and cartilage destruction. In RA, neoangiogenesis is an early and crucial event to promote the formation of pannus, causing further inflammatory cell infiltration. The transcription factor GATA4 is a critical regulator of cardiac differentiation-specific gene expression. We find that a higher level of GATA4 exists in synovium of rheumatoid arthritis (RA) patients, but the function of GATA4 in RA remains unclear. In the present study, IL-1β induces inflammation in fibroblast-like synoviocytes (FLS) MH7A, which is accompanied with the increased expression of GATA4 and VEGF production. Through application of GATA4 loss-of-function assays, we confirm the requirement of GATA4 expression for inflammation induced by IL-1β in FLS. In addition, we demonstrate for the first time that GATA4 plays key roles in regulating VEGF secretion from RA FLS to promote cellular proliferation, induce cell migration, and angiogenic tube formation of endothelial cells. GATA4 induces the angiogenic factors VEGFA and VEGFC, by directly binding to the promoter and enhancing transcription. The knockdown of GATA4 attenuates the development of collagen-induced arthritis (CIA) and prevents RA-augmented angiogenesis in vivo, which are accompanied with decreased VEGF level. These results reveal a previously unrecognized function for GATA4 as a regulator of RA angiogenesis and we provide experimental data validating the therapeutic target of GATA4 in RA mice.

## Introduction

As a chronic inflammatory disease, rheumatoid arthritis (RA) causes tissue damage by persistent inflammation^1^. Characteristics of this disease include degraded cartilage, moderate synovial inflammation, pain, and impaired mobility^2^. Several mechanisms contribute to the progression, including pathological angiogenesis^3^. Angiogenesis is the formation of blood vessels from pre-existing vasculature and tightly regulates physiological processes during normal growth, wound healing, and the female reproductive cycle^4^. However, angiogenesis can also play a detrimental role in various pathological conditions such as cancer, diabetic retinopathy, and chronic inflammatory diseases. In RA, angiogenesis occurs already in the earliest phases of the disease and is considered a switch from acute to chronic inflammation^5^. Chronic inflammation maintains blood vessel growth by the secretion of angiogenic factors by macrophages and other cells^6^, while synovial angiogenesis can further facilitate inflammation by increasing plasma extravasation and enhancing inflammatory cell recruitment^7,8^. Consequently, targeting angiogenesis might be considered as a new alternative in the treatment of RA^9^. For example, disease-modifying antirheumatic drugs (DMARDs) might affect synovial angiogenesis. As one of the DMARDs, methotrexate (MTX) is the anchor for treatment of RA with the abilities of inhibiting vascular endothelial cell proliferation as well as inflammation^7^. However, MTX failed to decrease VEGF levels in patients with RA^10^. Thus, further research is still required to identify another candidate molecules or pathways that could target angiogenesis for RA.

GATA4, a member of GATA zinc-finger transcription factor family, has been shown to regulate differentiation, growth, and survival of a wide range of cell types^11–13^. Recent evidence suggests that GATA4 mainly is abundantly expressed in cardiomyocytes throughout embryonic development, postnatal growth, and adulthood, during which it functions as a critical regulator of cardiac differentiation-specific gene expression^14^. In addition to regulating basal gene expression in the heart, GATA4 has been implicated as a mechanical load-responsive transcriptional mediator that can be activated by increases in cardiac afterload, vasopressin infusion, or through direct stretching of the ventricles in the isolated rat heart^15,16^. Heineke et al. first showed a novel function for GATA4 in the heart as a mediator of angiogenesis that facilitates compensation following injury^17^. They further demonstrated that overexpressed GATA4 in myocytes can induce angiogenesis in vitro through the secretion of angiogenic growth factors such as VEGF^18^. This paradigm of GATA4-dependent angiogenesis is also extended to a hind-limb ischemia model of injury, showing that GATA4 may serve as a global angiogenic regulator. However, little research has been done on the function of GATA4 in RA.

On the basis of these observations, we analyze the role of GATA4 in the modulation of angiogenesis and the pathogenesis of RA. Our researches identify that GATA4 is an attractive activator in the synovial lesions of patients with RA. Furthermore, the elevated levels of GATA4 contribute to inflammation and angiogenesis in RA. By gene loss-of-function assays, we demonstrate the angiogenesis is dependent on the GATA4 expression and VEGF secretion from RA fibroblast-like synoviocytes (FLS), the latter is also regulated by GATA4. We provide experimental data validating the therapeutic target of GATA4 in RA. This is a previously unrecognized function for GATA4 as a regulator of RA angiogenesis through disease-responsive mechanism.

## Results

### GATA4 and VEGF is upregulated in IL-1β-induced MH7A cells and in adjuvant-induced arthritis rat synovium

**Fig. 1:**
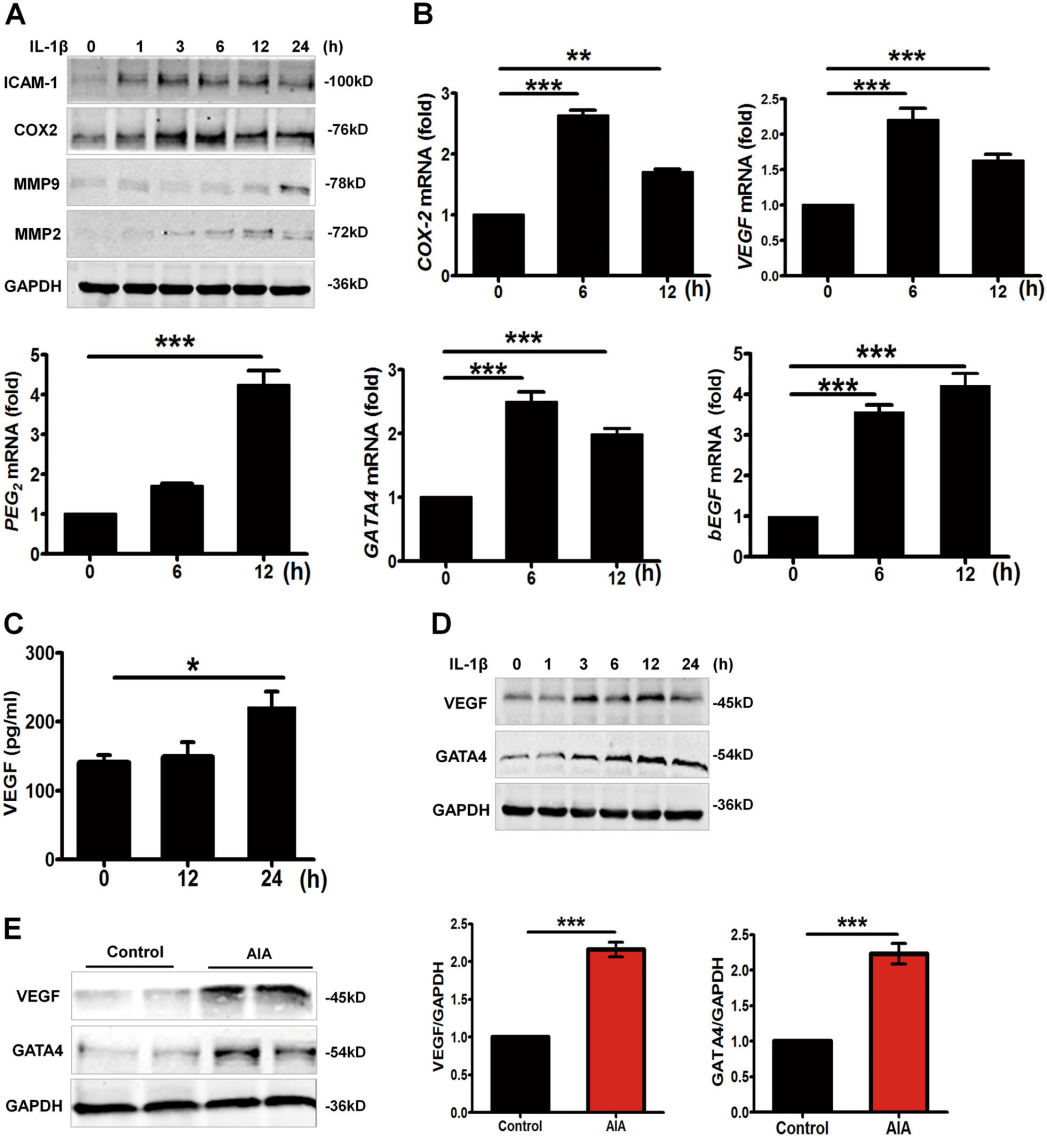
Increased GATA4 expression in IL-1β-induced MH7A cells and in synovial tissues of adjuvant-induced arthritis (AIA) rats. **a** MH7A cells were treated with IL-1β (10 ng/ml) for the indicated times, immunoblot analyzed for ICAM-1, COX2, MMP2, and MMP9. **b** mRNA levels of COX2, VEGF, PEG_2_ GATA4, and bEGF were examined by RT-qPCR in control and IL-1β-treated cells. **c** MH7A cells were starved with serum-free medium for 12 h, and then VEGF production was determined by ELISA assay in supernatant from control and IL-1β-treated cells. **d** Immunoblot was analyzed for VEGF and GATA4 for indicated time. All data are shown as mean ± S.E.M. from at least three replicates; ^*^p < 0.05, ^**^p < 0.01, ^***^p < 0.001. **e** Western blotting analysis showed the levels of VEGF and GATA4 of synovial tissue in control and AIA rats. Bars ± S.E.M. represent fold changes that are normalized to GAPDH and relative to controls (*n* = 6, ^***^p < 0.001)

IL-1β is a pivotal inflammatory cytokine that induces the generation of a range of additional inflammatory cytokines, which contributes to the progression of angiogenesis in RA. The MH7A synovial fibroblast cell line was employed in our experiment. This cell line was derived from immortalized synovial fibroblasts of a patient with RA using the SV40 T antigen^19^. As illustrated in Fig. 1a, IL-1β (10 ng/ml) dramatically increased inflammation markers in time-dependent manner, including ICAM-1, COX2, MMP2, and MMP9. Similarly, mRNA level of COX2 in IL-1β-stimulated MH7A cells were significantly increased (Fig. 1b). Next, we used real-time quantitative polymerase chain reaction (RT-qPCR) to examine mRNA level of GATA4 and angiogenic factors. Results showed that GATA4, VEGF, PEG_2_, and bEGF mRNA expression was significantly upregulated in MH7A treated by IL-1β for 6, 12 h (Fig. 1b). Soluble VEGF secreted in the supernatant of IL-1β-stimulating MH7A synovial cells were evaluated by ELISA kit (Fig. 1c). In addition, VEGF and GATA4 protein expressions were enhanced by incubating the cells with IL-1β in the time-dependent manner (Fig. 1d). To investigate the expression of GATA4 in synovium tissue, we conducted adjuvant-induced arthritis (AIA) rat model, one of the patterns that are characterized by the formation of pannus. As expected, the levels of GATA4 and VEGF were significantly increased in synovium of AIA rats groups in comparison with the control group (Fig. 1e). In summary, these data identified elevated pro-inflammatory factors, proangiogenic factors as well as GATA4 in IL-1β-induced FLS and RA.

### CM induces cells proliferation, migration, tube formation in HUVECs, and capillary sprouting in mouse aortic ring

**Fig. 2:**
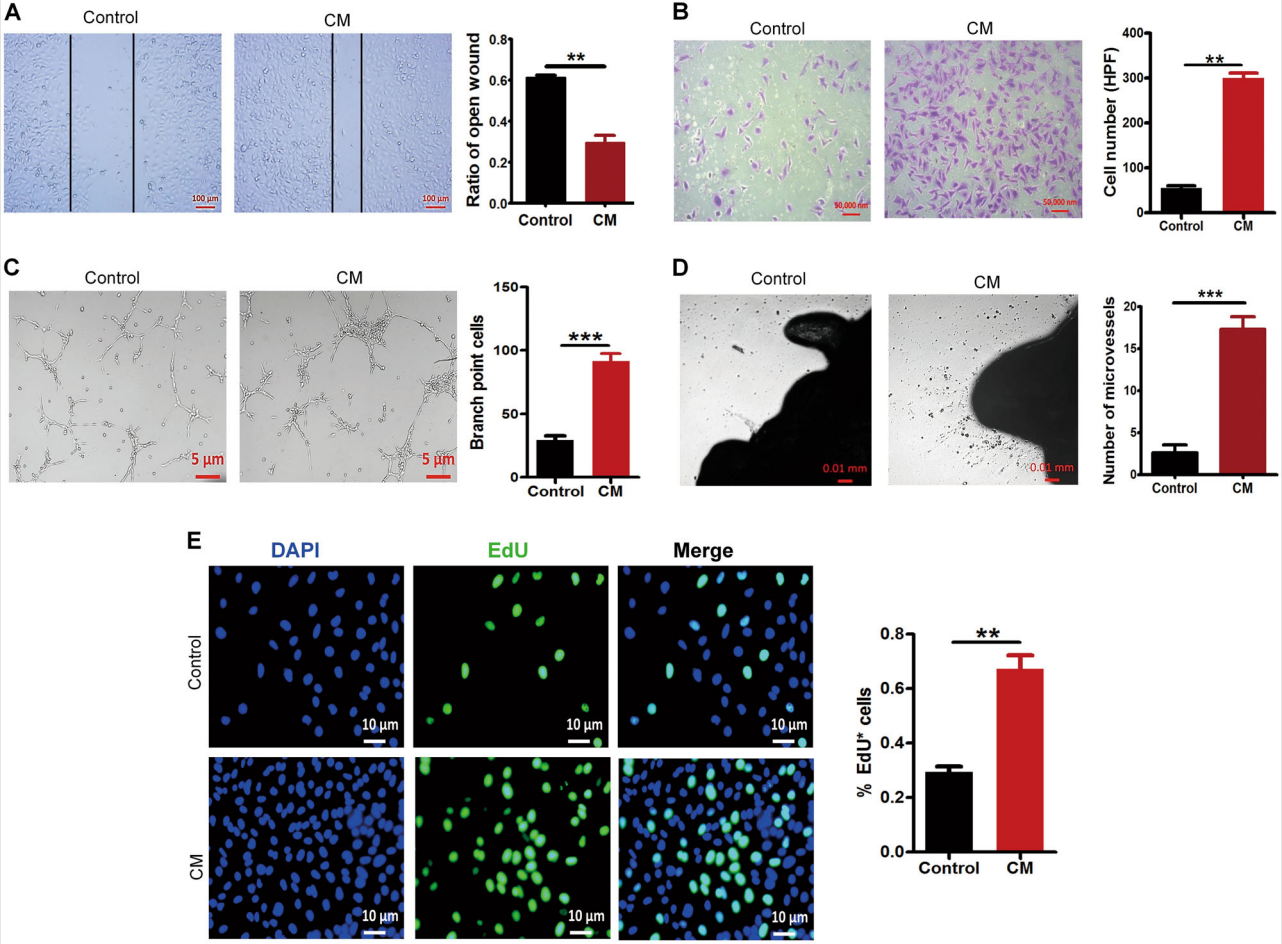
Conditioned medium (CM) induces cells proliferation, migration, tube formation in HUVECs, and capillary sprouting in mouse aortic ring. **a** HUVECs were treated with CM collected from MH7A cells pretreated with IL-1β (10 ng/ml) or not for 8 h, scratching assay was employed to examine the migration of HUVECs as described in Materials and Methods. **b** HUVECs were cultured in Boyden chamber, and the CM collected from MH7A cells treated by IL-1β or not were added in HUVECs culture medium for 24 h. The migrated cells were captured after fixation and crystal violet staining and quantified. Images were taken at ×100 magnification. **c** HUVECs were cultured for 4 h in the CM collected from MH7A cells that were pretreated with IL-1β, the branch point cells were determined and quantified. All data are mean ± S.E.M.; *n* = 6, ^**^p < 0.01; ^***^p < 0.001. **d** Aortic rings were embedded in Matrigel and cultured for 7 days in the presence of CM or not. **e** HUVECs were cultured for 24 h in CM collected from MH7A cells pretreated with IL-1β. EdU was employed to examine the proliferation of HUVECs and quantify analysis. All data are shown as mean ± S.E.M. from at least three replicates; ^**^p < 0.01, ^***^p < 0.001

CM (conditioned medium) was collected from MH7A induced by IL-1β for 24 h, and the pro-angiogenesis ability of CM was determined. Firstly, to analyze the proliferative effect of CM, CM was added to the HUVECs following scratching. After 8 h, the CM significantly accelerated the ECs migration (Fig. 2a). Similarly, transwell assay also showed that CM significantly increased EC invasion (Fig. 2b). In the tube-like structure formation assay, we found that CM significantly facilitated HUVECs’ tubular formation capacities after 4 h stimulation (Fig. 2c). Mouse aortic ring assay further verified the proangiogenesis capacity of CM (Fig. 2d). We also examined the effect of CM on the growth of HUVECs by EdU assay. As shown in Fig. 2e, CM from IL-1β-induced MH7A significantly increased proliferation of HUVECs. Therefore, we concluded that cytokines secreted by IL-1β-induced MH7A promoted EC proliferation, migration, tube formation, and mouse aortic ring capillary sprouting.

### Knockdown of GATA4 decreases VEGF generation and inflammatory mediators expression

**Fig. 3:**
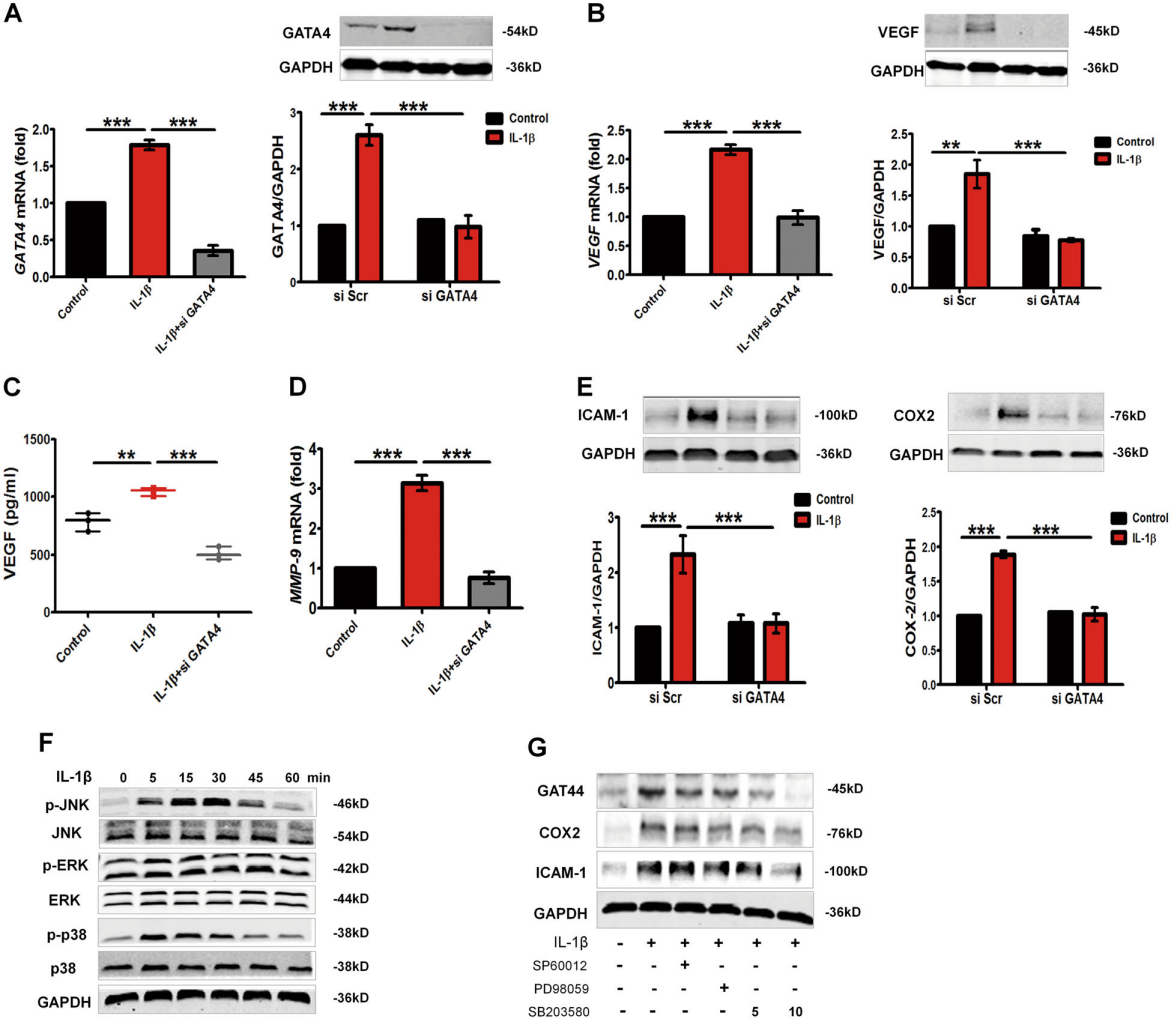
Knockdown of GATA4 reduces VEGF release and inflammation response in IL-1β-induced MH7A cells. **a** After MH7A cells were transfected with scramble siRNA (si Scr) or GATA4 small interfering RNA (si GATA4), they were treated with 10 ng/ml IL-1β for 12 h, qRT-PCR and western blotting was used to determine the expression of GATA4 (**a**) and VEGF (**b**); the level of VEGF production in supernatant was assessed by ELISA (**c**); mRNA level of MMP9 was determined by qRT-PCR (**d**); the expression of ICAM-1 and COX2 was determined by western blotting (**e**). All data are mean ± S.E.M. of three experiments; ^**^p < 0.01, ^***^p < 0.001. **f** IL-1β induced the phosphorylation of MAPKs in MH7A. FLS were treated with IL-1β (10 ng/ml) for indicated time, and cell lysates were prepared and blotted with total or phosphospecific antibodies to ERK1/2, p38, and JNK. **g** SB203580 (10 μM) inhibited GATA4 expression in IL-1β-induced MH7A. FLS were pretreated with SP60012, PD98059, or SB203580 (5 and 10 μM) for 4 h and then stimulated by IL-1β (10 ng/ml) for 24 h. The GATA4, ICAM, and COX2 were analyzed by western blotting, respectively

We further attempted to detect the association between GATA4 and VEGF generation in IL-β-induced MH7A. When MH7A cells were transfected with GATA4 siRNA, GATA4 expression was inhibited according to the results of RT-qPCR and western blot (Fig. 3a). Consistently, downregulation of GATA4 in MH7A cells decreased the mRNA and protein expression of VEGF with IL-1β treatment (Fig. 3b). As expected, VEGF level was concomitantly lower in the supernatant of MH7A cells transfected with GATA4-siRNA as compared to only IL-1β-induced MH7A cells by ELISA (510 ± 56 vs. 1045 ± 33 pg/ml, *p* < 0.001) (Fig. 3c). In addition, MMPs also regulate some molecules related to angiogenesis. Our results showed that downregulation of GATA4 in MH7A cells decreased the mRNA expression of MMP9 (Fig. 3d). Furthermore, knockdown of endogenous GATA4 decreased IL-1β-induced inflammatory mediators ICAM-1 and COX2 expression, whereas a scramble control siRNA (si Scr) did not have impact on their expression (Fig. 3e). Therefore, these results indicated that GATA4 might mediate IL-1β-triggered angiogenic factors in MH7A cells, and knockdown GATA4 could suppress the formation of angiogenic factors and inflammatory response.

As illustrated in Fig. 3f, IL-1β stimulation caused phosphorylation of MAPKs (JNK1/2, p38, and ERK1/2), eliciting a peak level of phosphorylated MAPK at various time in MH7A. And we found that IL-1β-induced GATA4 expression was suppressed by 10 μM SB203580 (a p38 inhibitor), whilst the expressions of ICAM-1 and COX2 were also restrained (Fig. 3g). These data suggested that MAPK pathway was responsible for the upregulation of GATA4 in IL-1β-stimulated MH7A.

### Conditioned medium from knockdown GATA4-MH7A (GM) inhibits cells proliferation, migration, tube formation in ECs, and capillary sprouting in mouse aortic ring

**Fig. 4:**
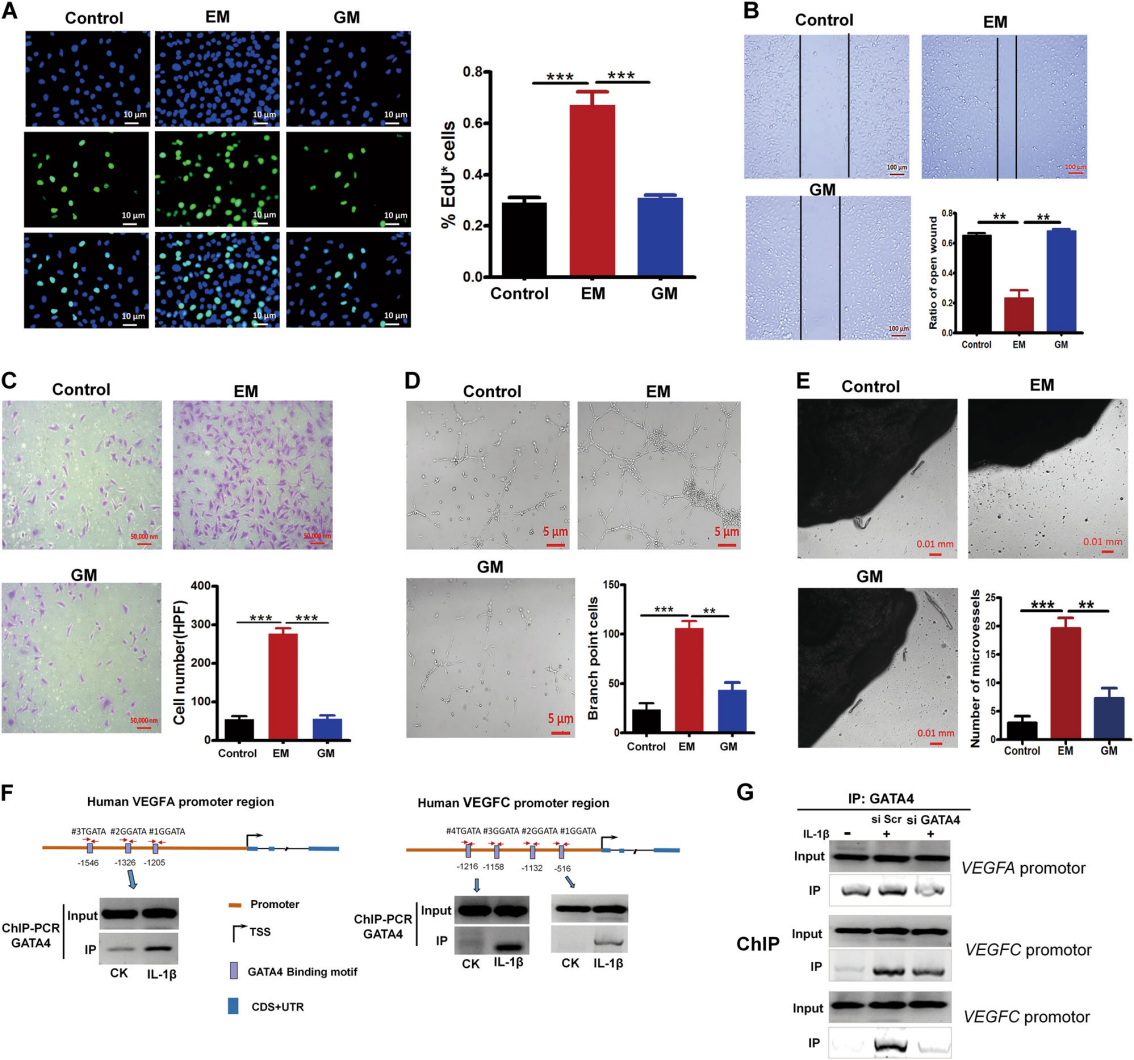
Conditioned medium from knockdown GATA4-MH7A (GM) inhibits cells proliferation, migration, tube formation in ECs, and capillary sprouting in mouse aortic ring. **a**–**d** MH7A cells were transfected with scramble siRNA (si Scr) or GATA4 small interfering RNA (si GATA4), and then treated with 10 ng/ml IL-1β for 24 h, conditioned medium were collected from si Scr (EM) or si GATA4 (GM). HUVECs were cultured with each conditioned medium for 24 h. EdU was employed to examine the proliferation of HUVECs (**a**); the scratching assay was employed to examine the migration of HUVECs as described in Materials and Methods (**b**); the migrated cells were captured and quantified after fixation and crystal violet staining as described in Materials and Methods (**c**); HUVECs were cultured for 4 h in the conditioned medium, the branch point cells was determined (**d**). Aortic rings were embedded in Matrigel and cultured for 7 days in the presence of control medium, EM and GM (**e**). All data are mean ± S.E.M. of three experiments; ^**^p < 0.01, ^***^p < 0.001. **f** The promoter region of rat *VEGFA* and *VEGFC* gene contains GATA4 binding motifs and IL-1β induction increased binding of GATA4 to motif regions by ChIP-PCR using GATA4 antibody. **g** Knockdown GATA4 decreased binding of GATA4 to motif regions upon IL-1β induction. MH7A cells transduced with si GATA4 were used for ChIP assay against GATA4 antibody

GATA4 suppression reduced VEGF production in IL-1β-induced MH7A cells, we thought this might decrease pro-angiogenesis ability. To test this hypothesis, we examined the proliferation, migration, and invasion of ECs treated by CM. We treated HUVECs with the CM collected from the IL-1β-induced MH7A cells that were transfected with scramble siRNA (EM) or with GATA4-siRNA medium (GM). The results showed that the number of EdU staining positive cells was ~3-fold lower in GM than in EM (Fig. 4a), suggesting the GM decreased proliferation effect on HUVECs. Then, we determined migration potential of EM and GM by wound assay. As shown in Fig. 4b, EM increased the migration of HUVECs, while GM treatment showed retarded migration. The transwell assay was performed simultaneously. Identically, GM from MH7A cell transfected with si GATA4 significantly inhibited invasion of HUVECs compared than EM (Fig. 4c). Moreover, in order to verify the effects of GATA4 on endothelial cell tube formation, the branch point cells of the tube-like structure in HUVECs treated by EM or GM, were observed after 4 h of culture. As indicated in Fig. 4d, the branch point cells cultured under EM were significantly increased compared to control conditions in HUVECs. This phenomenon was reversed by treatment with GM. The result in mouse aortic ring assay was in line with tube-like structure analysis. There were more microvessels in the rings treated with EM when compared with control group, while GM incubation reduced the number of microvessels (Fig. 4e). Together, these results suggested that GATA4 generated by IL-1β-induced MH7A cells promoted angeiogenesis, and knockdown GATA4 decreased pro-angiogenesis ability.

### GATA4 binding to *VEGFA* and *VEGFC’s* promoter enhances transcription in IL-1β-induced FLS

To investigate the mechanism by which GATA4 enhanced pro-angiogenesis ability, we analyzed the promoter sequence of *VEGFA* and *VEGFC* gene and found that GATA4 could be a potential upstream transcription factor. Three and four DNA-binding motifs of GATA4 were identified within 2 kilobyte (kb) upstream of Transcription Start Site (TSS) of *VEGFA* and *VEGFC*, respectively (Fig. 4f). Using chromatin immunoprecipitation followed by polymerase chain reaction (ChIP-PCR) assay, IL-1β-treated cells showed enhanced GATA4 binding to the *VEGFA* and *VEGFC’ s* promoter region when compared with untreated cells (CK) (Fig. 4f). To further validate the regulatory role of GATA4 on VEGF, GATA4 was knocked down by siRNA. The result showed that depletion of GATA4 led to decreased GATA4 binding to the *VEGFA* and *VEGFC’ s* promoter region when compared with IL-1β-treated cells (Fig. 4g). These results suggested that upregulation of VEGF in IL-1β-induced cells was mediated by GATA4-dependent mechanism, in agreement with previous studies reporting that GATA4 directly binds to *VEGF’s* promoter in regulatory of heart angiogenesis.

### Knockdown GATA4 reduces collagen-induced arthritis incidence and severity

**Fig. 5:**
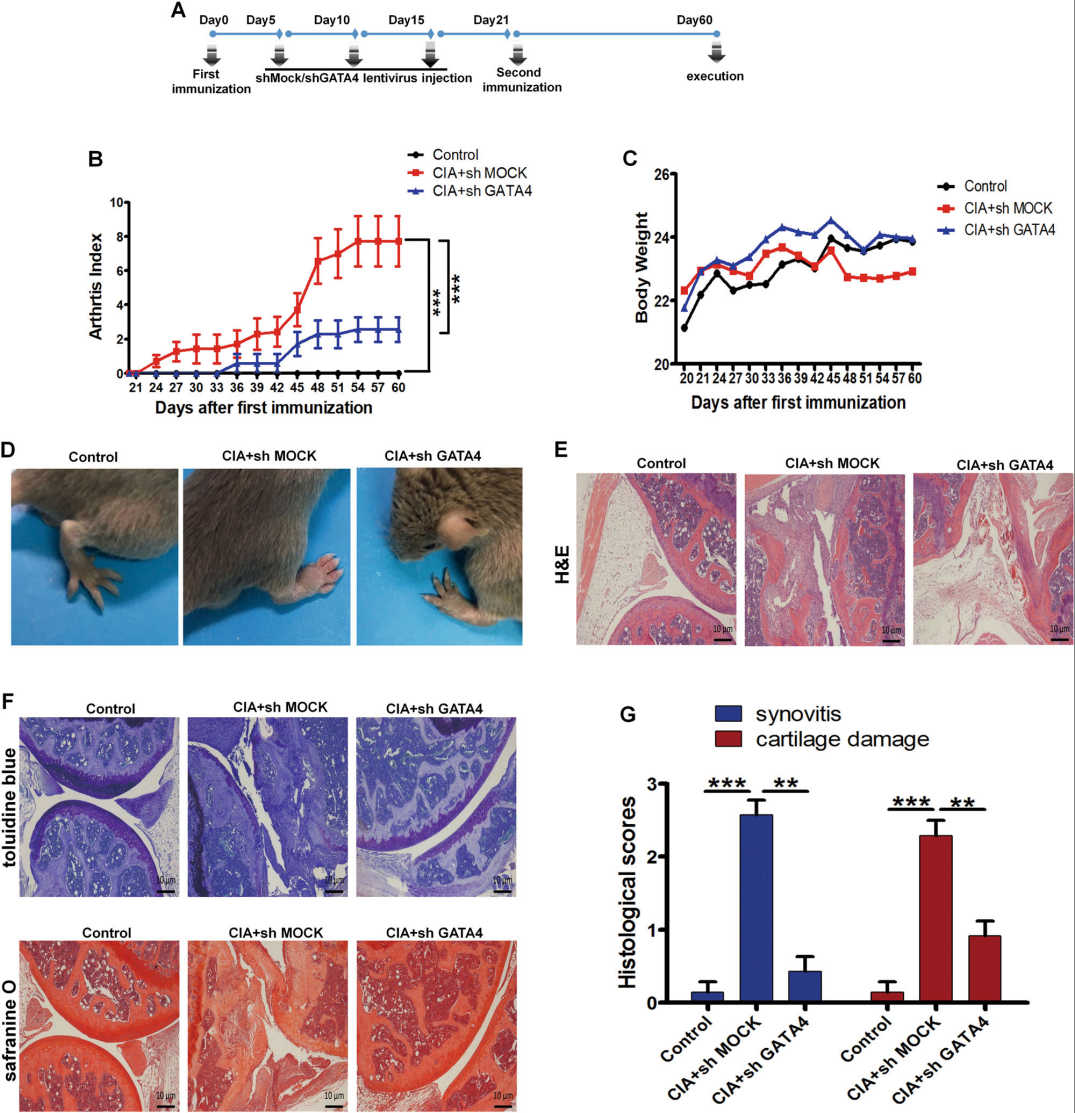
Knockdown of GATA4 reduces severity of arthritis in collagen-induced arthritis (CIA) mice. **a** Schematic drawing of experimental setup. Mice received injection of either 150 μl lentivirus shGATA4 or vector (sh MOCK) every 5 days beginning 5 days before the initial immunization. **b** Knockdown of GATA4 significantly decreased the mean arthritis score and increased the time of arthritis first appeared compared with sh MOCK-treated CIA mice. Data are mean ± S.E.M., *n* = 10; ***p < 0.001. **c** Body weight of Control, shMOCK, and shGATA4 mice with CIA during 40 days post second immunization was recorded. **d** Macroscopic evidence of arthritis such as erythema or swelling was markedly observed in shMOCK-treated CIA mice, while knockdown of GATA4 with lentivirus shGATA4 significantly attenuated arthritis severity in CIA mice. **e** Representative H&E staining of knee joint sections of Control, shMOCK, and shGATA4 mice with CIA. **f** Representative toluidine blue and safranin O staining of knee joint sections of Control, shMOCK, and shGATA4 mice with CIA. **g** The quantitative analysis for synovitis and cartilage damage from Control, shMOCK, and shGATA4 mice with CIA. Data are mean ± S.E.M., *n* = 6; **p < 0.01; ***p < 0.001

Having demonstrated the potential of GATA4 in mediating inflammatory cytokines and angiogenesis, we next validated this effect in an in vivo model with lentivirus-mediated knockdown of GATA4. The collagen-induced arthritis (CIA) model shares many pathological features with human RA, including synovial hyperplasia, joint swelling, cartilage destruction, and vessel angiogenesis. Genetically susceptible DBA/1J mice were immunized with Chick type II collagen (CII) emulsified in Freund adjuvant (CFA), 5 days after the initial immunization the mice were injected with vector lentivirus control (sh MOCK) or encoding GATA4 short-hairpin RNA (sh GATA4). Figure 5a showed time schedule for the animal experiment using sh GATA4 or sh MOCK injection. The similar age and gender mice were taken as normal controls. The assessment for clinical symptoms was conducted every third day from day 21 until day 60. In sh MOCK mice with CIA, symptoms started to appear from day 24 and reached a score of >2 per animal by day 36. However, CIA mice treated with sh GATA4 developed less severity and lower incidence of arthritis as determined by clinical scores (Fig. 5b,c). Images of mice hindpaws showed significant reduction in inflammation and soft tissue swelling upon treatment with sh GATA4 (Fig. 5d). Mice were sacrificed on day 60 for histopathological examination of joints. Reduced severity of arthritis in sh GATA4-treated mice was associated with decreased infiltration of immune cells in affected joints (Fig. 5e). Histological analysis also revealed reduced subchondral bone erosions in shGATA4 mice. Knockdown GATA4 protected the collagen-immunized animals from severe bone destruction as seen in representative histomorphometric measurements (Fig. 5f, g). These data indicated that knockdown GATA4 reduced clinical symptoms, joint pathology, and local bone destruction in CIA mice.

**Fig. 6:**
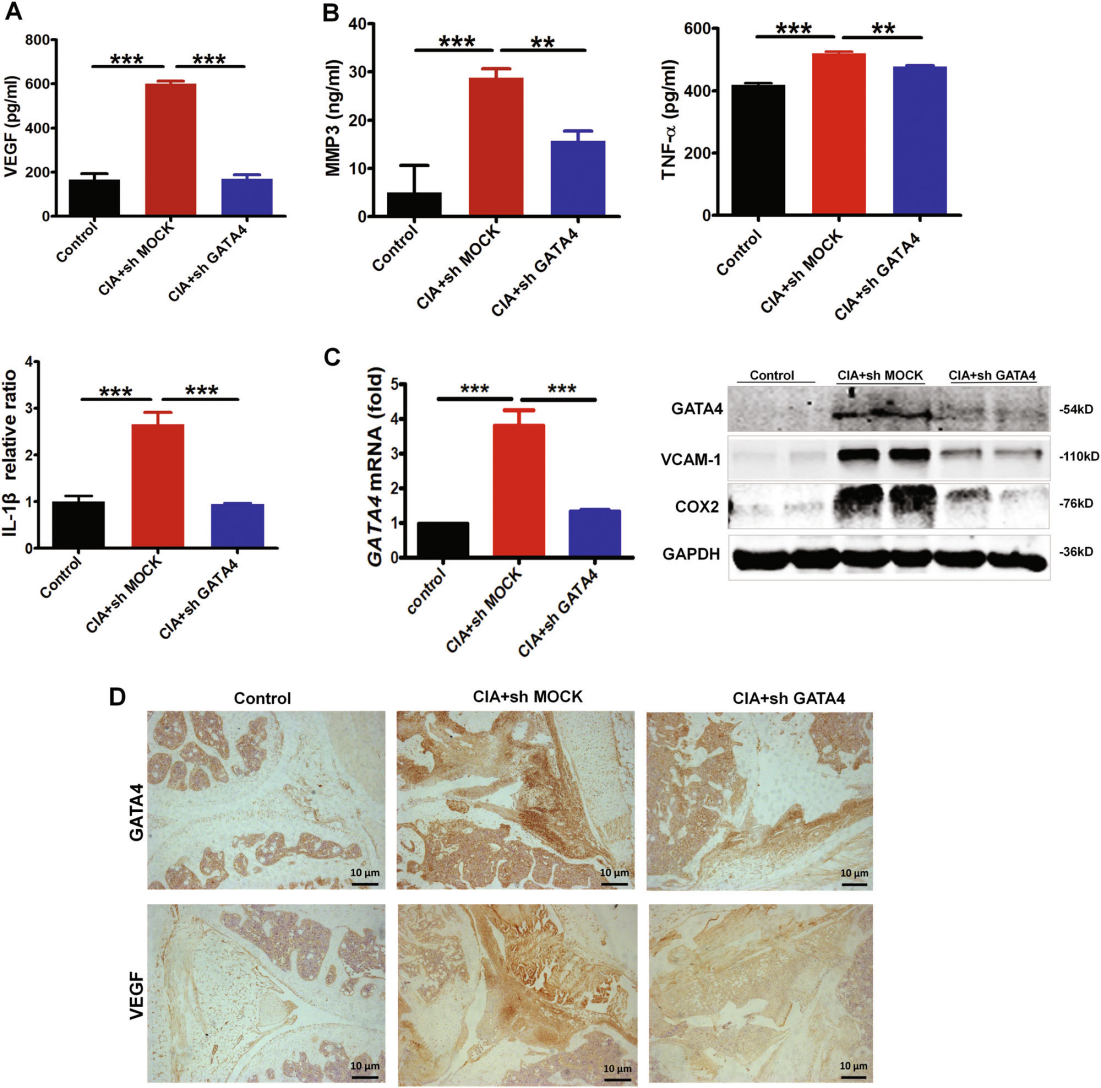
Blocking GATA4 inhibits VEGF production and joint inflammation in collagen-induced arthritis mice. **a** Mean circulating serum VEGF level in Control, shMOCK, and shGATA4 mice with CIA was measured at day 60 after first immunization. **b** Mean circulating serum inflammation cytokines MMP3, TNF-α, and IL-1β levels in Control, shMOCK, and shGATA4 mice with CIA were measured at day 60 after first immunization. **c** GATA4 mRNA level and GATA4, VCAM-1, and COX2 protein levels in synovial tissues from Control, shMOCK, and shGATA4 mice with CIA were detected by qRT-PCR and western blotting. **d** Representative images of immunohistochemistry microscopy of GATA4 and VEGF in knee joints sections of Control, shMOCK, and shGATA4 mice with CIA. Data are mean ± S.E.M., *n* = 6; **p < 0.01; ***p < 0.001

Furthermore, the levels of the VEGF and pro-inflammatory cytokines in serum were measured by ELISA at the termination of the experiment. As expected, the serum level of VEGF in the CIA mice was significantly increased, whereas knockdown GATA4 exhibited a dramatic decrease (Fig. 6a). Consistent with joint swelling, knockdown GATA4 exhibited a dramatic decrease in TNF-α, MMP3, and IL-1β cytokines (Fig. 6b). Next, we analyzed the mRNA level of GATA4 in joints of the mice and found the successful knockdown of GATA4 when treated with sh GATA4 (Fig. 6c). Protein level of GATA4 in synovium showed the same efficiency, and blocking GATA4 also down-regulated the expression of VCAM-1 and COX2 (Fig. 6c). Histological examinations also indicated that blocking GATA4 suppressed VEGF expression (Fig. 6d). Those suggested that knockdown of GATA4 resulted in the amelioration of joint inflammation, erosion and angiogenesis.

### Evidence of GATA4 upregulation in human RA synovialcytes

**Fig. 7:**
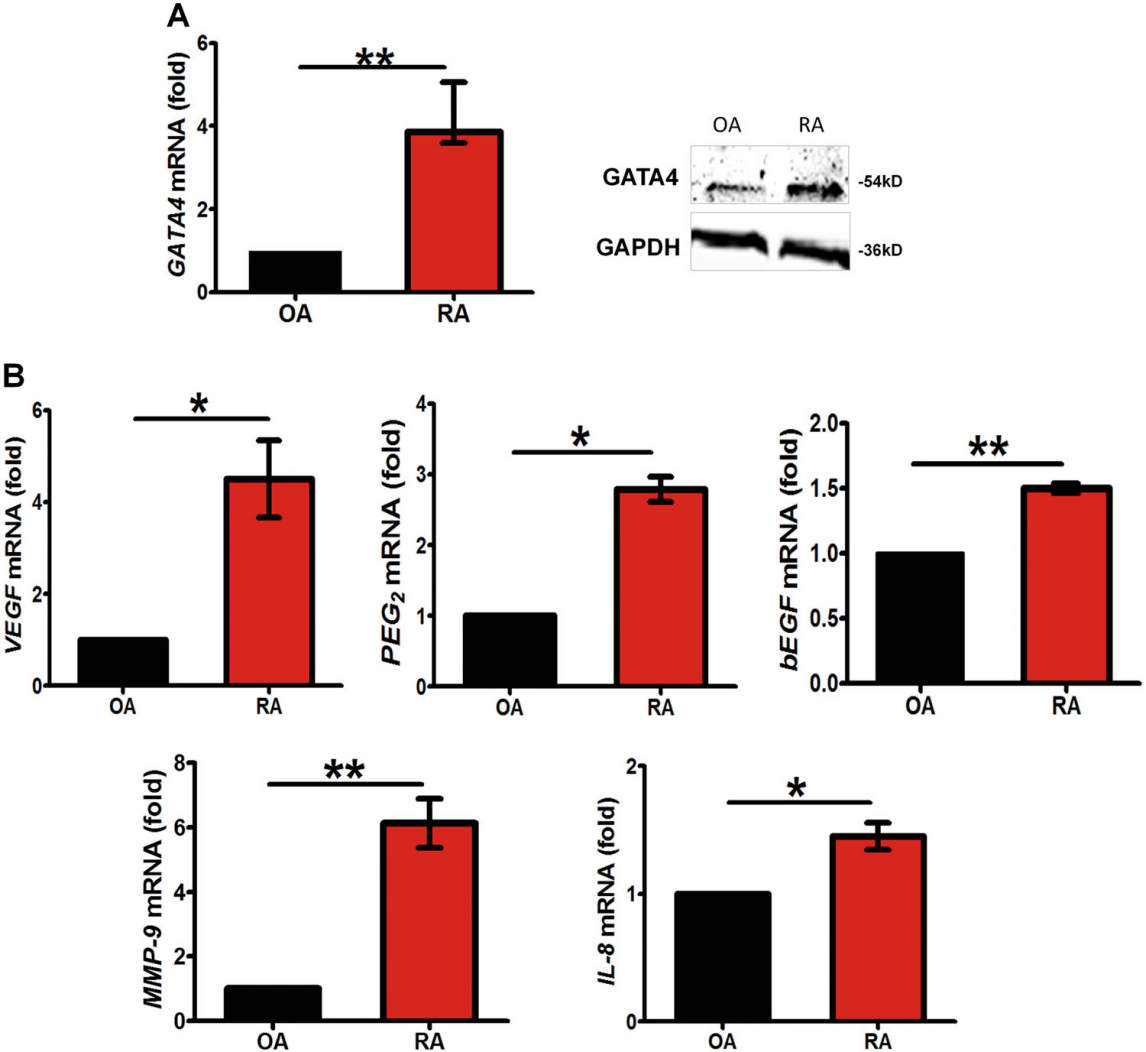
GATA4 expression is increased in FLS from RA patients. Cell extracts from six OA and RA patients’ synovial tissues were assessed with qRT-PCR or western blot. The expression of GATA4 mRNA and protein in FLS from RA or OA patients’ synovium was determined (**a**). Expression of VEGF, bEGF, PEG_2_, MMP9, and IL-8 mRNA in FLS from RA or OA patient’s synovium (**b**). Data are mean ± S.E.M.; *n* = 6, *p < 0.05; **p < 0.01. RA rheumatoid arthritis, OA osteoarthritis

RT-qPCR and western blotting analysis was performed to determine the expression of GATA4 in synoviocytes from osteoarthritis (OA) and RA patients. The results showed that, compared to OA, RA exhibited the increased GATA4 mRNA and protein levels (Fig. 7a). As expected, mRNA expression of VEGF, bEGF, and PEG2 in the RA synoviocyte samples were higher than in the OA samples. The expression of inflammatory mediators MMP9 and IL-8 consistently were increased compared with OA (Fig. 7b). These data raised the possibility that increased GATA4 expression may be implicated in the pathogenesis of RA.

## Discussion

In RA, neoangiogenesis is an early and crucial event to promote the development of the hyperplasic proliferative pathologic synovium and foster inflammation^9,20^. Emerging evidence implicates that targeting synovial angiogenesis may provide a promising therapeutic approach for RA^21^. Recently, the transcription factor GATA4 is reported as a regulator of angiogenesis in the heart^17,22^, but the function of GATA4 in RA, and the relationships between GATA4 and RA pathology remains unclear. Thus, we hypothesized that GATA4 promoted the angiogenesis in the RA. Consistently, we found that GATA4 expression was elevated in synovialcytes derived from RA patients and in synovium from AIA rats. We demonstrated that knockdown GATA4 decreased the production of pro-inflammatory cytokines and angiogenic factors in IL-1β-induced MH7A. Furthermore, we also confirmed that CM from knockdown GATA4-MH7A inhibited the migration, invasion and proliferation of ECs, reduced the tube formation in vitro. The underlying mechanism involved in overexpressed GATA4 directly binding to the *VEGFA* and *VEGFC’s* promoter and enhancing transcription, which promoted ECs migration and proliferation, launching the vascular stage of the disease. Knockdown GATA4 in vivo markedly reduced the severity of synovial hyperplasia, inflammatory cell infiltration and joint destruction in mice with CIA. Our findings strongly suggested the elevated GATA4 expression promoted angiogenesis and maintained inflammation in RA consequently.

Inflammation and angiogenesis play a central role in many autoimmune diseases including RA. Mediators of inflammation have a significant effect on the process of angiogenesis^23,24^, and the reverse is also true^25,26^. RA FLSs are implicated in the inflammatory response essentially by synthesizing cytokines, chemokines, and proangiogenic factors^27^. Angiogenesis, which is involved in the regulation of several soluble and cell surface-bound factors, plays a central role in the RA pathogenesis for a long time including synovial hyperplasia and progressive bone destruction^28,29^. GATA4, as a zinc-finger transcription factor, binds specifically to the HGATAR-containing DNA motifs through their highly conserved zinc-finger DNA-binding domains^30^. It proves to be a cardiac-specific transcriptional activator. Previous studies have pointed out that, in addition to its important role in cardiac physiology and pathology, GATA4 has also been reported to regulate transcription in the gut, lungs, and ovaries ^31–34^. In spite of the important function of GATA4 in heart development and homeostasis, the role of GATA4 in RA remains unknown. In this study we found GATA4 expression was increased significantly in synovialcytes from RA patients. We demonstrated that the upregulated expression of GATA4 also presented in synovial tissues from mice with CIA, rats with AIA, and in IL-1β-induced FLSs. We further confirmed that IL-1β induced GATA4 expression in FLSs, whose conditioned medium promoted angiogenic tube formation, cell proliferation, and cell migration of HUVECs. The expression of GATA4 is required for all of these phenotypes induced by IL-1β. Importantly, knockdown of GATA4 by siRNA completely inhibited GATA4-induced capillary formation in vitro. Although, we demonstrated for the first time that GATA4 plays an important role in the angiogenesis in RA, it is consistent with report that GATA4 functions as a stress-responsive regulator of angiogenesis in the murine heart^18^.

Moreover, since knockdown GATA4 in MH7A cells inhibited the production of inflammation mediators such as iNOS and COX2 induced by IL-1β, which indicated that GATA4 played a global role in inflammatory response by regulating the expression or release of inflammation mediators in RA. The treatment with conditioned medium of IL-1β-induced FLSs transfected siGATA4 could inhibit angiogenic ability of ECs, indicating that GATA4 also mediated the interaction between RA-FLSs and its surrounding cells. During RA, angiogenesis is increased in the subchondral bone, which leads to vascularization and lesion formation in the RA cartilage^35^. These in vitro results were supported by in vivo experiments, showing that knockdown GATA4 attenuated synovial inflammation and joint destruction in mice with CIA. Thus, our findings further supported the notion that increased synovial GATA4 expression contributes to joint inflammation and angiogenesis in RA.

VEGF is the main signaling protein involved in angiogenesis; it is detected in synovial tissue and fluid of RA, as well as in serum. The VEGF expression level in synovial fluid and tissues correlates with the clinical severity of human RA and the degree of joint destruction^36^. During RA, VEGF exerts its effects through binding to receptors and promote endothelial migration and proliferation to form vascular tubules^20,37^. Based on the role of angiogenesis during pathogenesis of RA, inhibition of joint neovascularization may be more effective in controlling synovitis and joint destruction. The dual activities of VEGF as an endothelial-cell mitogen and a modulator of changes in vascular permeability are of relevance in the pathogenesis of RA. In this study, we found that increased GTAT4 expression in the synovial tissues of RA may contribute to the abnormal VEGF expression as evidenced by western blot and ELISA assay. Furthermore, we believed that induction of this program may be straightforward, since the *VEGFA* and *VEGFC* genes are binding targets for GATA4 and given the reciprocal regulation of angiogenesis that occurred in GATA4 gain- and loss-of-function approaches in FLSs and CIA mouse. Importantly, knockdown of GATA4 by siRNA completely inhibited *VEGFA* and *VEGFC* gene binding to GATA4, indicating that induction of GATA4 was an essential part of angiogenic gene program in RA. Therefore, GATA4 can directly regulate VEGF expression and the angiogenic program in the RA. These results also highlighted that GATA4 promoted angiogenesis following pathologic stimulation, suggesting a possible novel therapeutic approach to inhibiting the angiogenic program by GATA4 target or by other means of decreasing its activity.

**Fig. 8:**
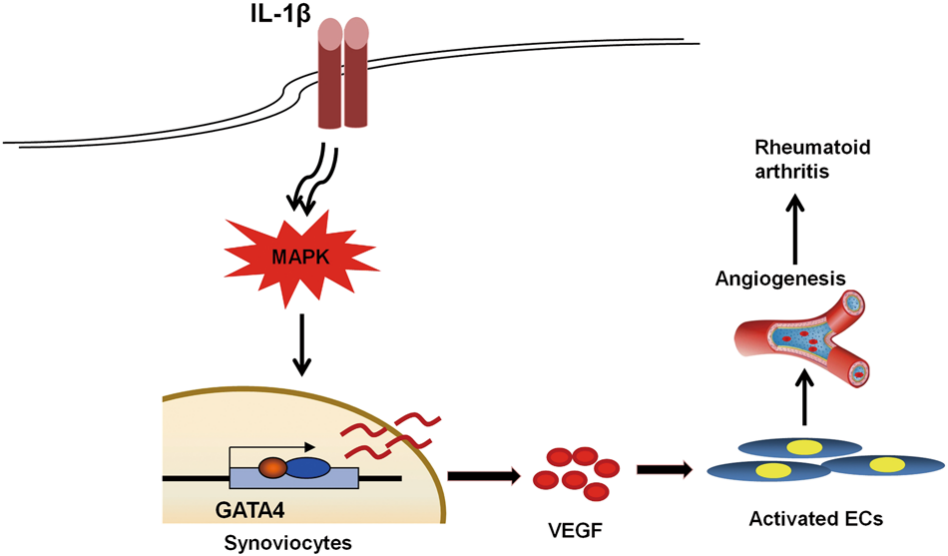
Working model for GATA4 regulates angiogenesis and persistence of inflammation in rheumatoid arthritis. FLS has lower level of GATA4 protein. After IL-1β induced FLS or in CIA mice, GATA4 level is increased. As a consequence, GATA4 binds to the promoter of VEGF gene and enhances the transcription of VEGF, causing angiogenesis and persistence of inflammation in rheumatoid arthritis

In summary, we demonstrated the increased GATA4 expression from RA FLSs partly mediated the angiogenesis pathology process in RA. In addition, IL-1β induced the GATA4 expression in FLSs, which directly bind on promotors of *VEGFA* and *VEGFC* to promote transcription, further regulated the angiogenesis in RA (see working model in Fig. 8). Taken together, our data pointed toward GATA4 as a novel therapeutic target in RA.

## Materials and methods

### Reagents

Reagent sources were as follow: Chick type II collagen (CII) and Freund adjuvant (CFA) were obtained from Chondrex (CII; Chondrex, Remond, WA, USA). The p38 inhibitor SB203580, the JNK inhibitor SP60012 and the ERK inhibitor PD98059 were purchased from Calbiochem (San Diego, CA). Antibodies COX2, GATA4, GAPDH were obtained from Santa Cruz Biotech (Santa Cruz, CA); VEGF (ab46154) was purchased from Abcam; ICAM-1 was purchased from Proteintech (Proteintech, USA). Antibodies phosphor (p)-ERK1/2 (Thr202/Tyr204) (9101), ERK1/2 (9102), p-JNK (Thr183/Tyr185) (9251), JNK (9252), p-p38 (9216), p38 (9212) were purchased from Cell Signaling technology (Beverly, MA).

### Ethic statement

Male Sprague-Dawley (SD) rats weighing 200–220 g and Male DBA/1 mice (6–8 weeks) were purchased from SHANGHAI SLAC LABORATORY ANIMAL CO. LTD (Shanghai, China). All animals were housed under conventional conditions and the experimental protocol conformed to the Animal Welfare Act Guide for Use and Care of Laboratory Animals, and was approved by Institutional Animal Care and Use Committee (IACUC), School of Pharmacy, Fudan University, China.

Human synovial samples were obtained from the knee joints of six patients with RA treated in Guanghua Integrative Medicine Hospital, Shanghai, China. Control synovial samples from six patients with osteoarthritis (OA) were obtained from Ningbo The 9th Hospital, Ningbo, China. All patient samples were taken with patient consent and full local regional ethics committee.

### Cell culture and collection of CM

We thank Professor Zhang Peng (Chinese Academy of Science, Shenzhen) for providing FLS MH7A cell lines, which were purchased from the Riken cell bank (Tsukuba, Japan). MH7A cells were cultured in Dulbecco’s modified Eagle’s medium (DMEM) in high glucose, supplemented with 10% fetal bovine serum (Hyclone) and 1% penicillin/streptomycin, in a 5% CO_2_ humidified atmosphere at 37 °C, and human umbilical vein endothelial cells (HUVECs) were cultured in endothelial cell growth medium (ECM, ScienCell, California, USA) supplemented with 10% fetal bovine serum. MH7A cells at passages 4–6 were used, and HUVECs at passages 3–5 were used in our experiments.

MH7A cells were treated or not (control) with 10 ng/ml of IL-1β (Peprotech, Rocky Hill, NJ, USA) for 24 h. At the end of stimulation, supernatants were collected and centrifugated (5 min, 1600 rpm) to remove cells and debris as CM for further use. EM and GM were collected from the culture medium of IL-1β-induced MH7A cells transfected with scramble control siRNA (siScr) and GATA4 siRNA (si GATA4), respectively.

### Real-time quantitative polymerase chain reaction

Total RNA was isolated with the use of TRIzol (Invitrogen, Carlsbad, CA, USA) according to the manufacturer’s instruction, and then reversely transcribed into cDNA using the Primer Script RT reagent Kit (TaKaRa Biotechnology Co., Ltd., Dalian, China). The PCR outcome was determined using CFX96 Real-Time PCR Detection System (Bio-Rad). The primers in RT-qPCR reaction were used as Table S1.

### Western blot assay

Cells or synovial tissue were lysed to quantitate protein levels using BCA protein assay (ThermoFisher Scientific). Equal amounts (25 mg) of proteins were separated and transferred to nitrocellulose membrane. Specific primary antibodies used included, then visualized by secondary antibodies conjugated with horseradish peroxidase. Immunoreactive proteins were visualized by enhanced chemiluminescence and signal intensity was detected and quantified by Alpha Imager (Alpha Innotech Corp, San Leandro, CA).

### Chromatin immunoprecipitation PCR

ChIP assays of MH7A cells induced by IL-1β (10 ng/ml) for 6 h were performed. The cells were crosslinked with final concentration 1% formaldehyde for 10 min at room temperature. Then 125 mM glycine was added to quench unreacted formaldehyde. The cells were gathered and sonicated to make DNA fragments with a size range of 200–1000 bp. The cell extracts were immunoprecipitated using 2 μg anti-GATA4 for each sample suspended in 300 μl Chromatin buffer (1× protease inhibitor, 1.0% Triton X-100, 0.5 mM EGTA, 1.0 mM EDTA, 10 mM Tris-HCl, pH 8.0). For all ChIP experiments, PCR analysis was performed by using multiple sets of primers spanning the transcription factor binding site on *VEGFA* and *VEGFC* gene promoter. Sequences of primers for the ChIP assay were as follows: VEGFA: 5′-CAAAGAGGGAACGGCTCTCA-3′ (forward) and 5′-AAAATTACCCATCCGCCCCC-3′ (reverse). VEGFC-1: 5′-CTCCGAGGTCCCATAGGGTT-3′ (forward) and 5′-GCTTTATCCTCGGCCACTCC-3′ (reverse); VEGFC-2: 5′- ATCACCTCTAAAGCCGGTCC-3′ (forward) and 5′-GCTTATGTGAGAGAAAGCGGC-3′ (reverse).

### Endothelial cell proliferation assays

The HUVECs proliferation was measured by performing 5-ethynyl-2′-deoxyuridine (EdU) incorporation assay, using EdU assay kit (KeyGEN BioTECH, Shanghai, China), according to the manufacturers’ instructions. Briefly, 5 × 10^3^ cells per well were plated in 24-well plates for 12 h, then incubate with CM for 24 h at 37 °C. Fifty µM of EdU was added to each well and cells were cultured for additional 4 h at 37 °C, then the cells were stained with DAPI and visualized under a fluorescent microscope (Zeiss LSM780, Carl Zeiss). The proliferative cells were stained with green color and the EdU incorporation rate was expressed as the ratio of EdU positive cells to total DAPI positive cells (blue cells).

### Endothelial cell invasion and migration assay

The transwell assay was applied to assess the invasion effect of MH7A-secreted factors to the endothelial cells. HUVECs suspended in 0.2 ml of serum-free ECM at 2 × 10^4^ cells/well to the 24-well upper chamber, and 0.8 ml of DMEM filled with CM, which served as the chemoattractant, was added to the bottom well. After incubation for 24 h, the transwell inserts were fixed with 4% paraformaldehyde for 10 min and stained with 0.05% crystal violet. The migrated cells on the bottom layer were captured and measured by counting the number from five randomly chosen fields under a light microscope (Zeiss LSM780, Carl Zeiss).

The scratch assay was conducted to measure cell migration. HUVECs, plated to confluence on 35-mm culture dishes, were wounded with 1-ml sterile micropipette and then washed three times with starving media to remove detached cells. The remaining cells were treated with or without CM. After 8 h of incubation, migration was detective by the distance that the cells that moved beyond a reference line. The wound width and area were analyzed using Digimizer.

### Tube formation assay

HUVECs were pre-cultured overnight in ECM with 0.25% FBS and then reseeded at a density of 2.5 × 10^4^ cells/well in 48-well plates pre-coated with Matrigel Matrix (BD Biosciences). HUVECs were treated with 0.25% FBS containing CM or not for 4 h. After that, the capillary-like tube formation of each well in the culture plates was photographed (Zeiss LSM780, Carl Zeiss). These images were analyzed using NIS Elements AR Analysis 4.10.00 (Nikon, Tokyo, Japan).

### Mouse aortic ring assay

48-well plates were covered with 200 μl Matrigel and incubated at 37 °C in 5% CO_2_ for 30 min. The thoracic aortas were isolated from C57 mice with the periadvtitial fat and connective tissues cleaned. After washed with PBS, thoracic aortas were cut into 1 mm long rings, placed in the Matrigel-covered wells. And then another 200 μl Matrigel was added into the aortas rings. And then another 200 μl Matrigel was added into the aortas rings. Once the upon-layer matrigel solidified, 200 μl CM was added into the well. After 7 days, the microvessel growth was photographed (Zeiss LSM780, Carl Zeiss). The number of microvessels was measured by using Adobe Photoshop software.

### Induction of rat AIA

Arthritis was induced by Freund’s complete adjuvant (CFA) inoculation of the SD rats. Briefly, on day 0, rats were anesthetized and then injected intradermally at the base of the tail with 0.05 ml CFA containing 10 mg/ml of heat-inactivated Mycobacterium (Catalog #7027, Chondrex, Inc). Rats in the control groups were injected with an equal volume of saline instead of CFA.

### Induction of mice CIA

DBA/1 mice were immunized intradermally with chicken type II collagen emulsified with CFA, administered at the tail base on day 0, and received booster injections of emulsion on day 21 with chicken type II collagen emulsified in IFA, resulting in a more than 90% incidence of arthritis, as previously reported^38^.

### Lentivirus generation and in vivo shRNA delivery

The lentivirus vectors were produced by cotransfection of 293T cells with the shRNA GATA4 plasmids (Obio technology, Shanghai) along with the packaging plasmid psPAX2 and the envelope plasmid pMD2.G. shRNA GATA4 lentivirus were harvested and concentrated by ultracentrifugation. CIA mice were injected via tail vein purified lentiviral particles (1 × 10^8^ MOI) that carry short-hairpin RNA targeting GATA4 (sh GATA4) every 5 days from day 5 after the first CII immunization to day 15. Control CIA mice were injected via tail vein vector lentiviral particles (sh MOCK).

### Evaluation of arthritis

All four paws of the CIA mice were examined by a blinded observer every 3rd day for the signs of swelling and/or redness and evaluated and scored from 0 (no inflammation) to 4. 0 = no disease; 1 = mild swelling or redness of just one group of joints; 2 = swelling and redness of one whole foot which can move normally; 3 = moderate swelling and redness of one whole foot whose moving ability is partly limited; 4 = the joints are severe swelling, redness, and/or ankylosis, and the limb cannot move at all^39^.

### Histological analysis of joint tissue and immunostaining

Mouse joint tissues were decalcified with 0.5 M ethylenediamine tetraacetic acid (EDTA, pH 8.0) for 2 weeks, embedded in paraffin, and sectioned at 5 μm thickness. Synovitis was evaluated via hematoxylin and eosin staining of joint sections, which was scored from 0 to 3 defined as hypercellularity of the synovium, including pannus formation. And the destruction of cartilage, which was defined as the loss of aggrecan, as measured by Safranin O or toluidine blue staining of articular cartilage (0 = fully stained cartilage, 3 = totally unstained cartilage).

### Immunohistochemical staining

Tissue sections were prepared as described above. Endogenous peroxidase activity was inhibited by 3% hydrogen peroxide. Antigen retrieval was performed by autoclaving at 120 °C for 15 min in 0.01 M citrate buffer (pH 6.0). The sections were reacted overnight with the following rabbit polyclonal antibodies: anti-VEGF (diluted 1:200) and anti-GATA4 (diluted 1:150) at 4 °C. The secondary antibody biotinylated anti-rabbit IgG was applied for 30 min at room temperature. The sections were visualized by 3, 30-diaminobenzidine-tetrahy-drochloride (DAB).

### Cytokine measurement

VEGF, TNF-α, IL-1β, and MMP3 levels in supernatant or serum were measured by using commercially available Enzyme-linked immunosorbent (ELISA) kits (Boatman BioTech, Shanghai, China), according to the manufacturer’s instructions.

### Establishment of human FLS

Synovial tissues were isolated from patients with RA and OA at the time of arthroscopic biopsy or total joint replacement. Synovial tissue specimens were minced into small pieces and treated with 1 mg/ml of collagenase (Sigma) for 2 h at 37 °C in serum-free RPMI 1640, filtered through a nylon mesh, and washed extensively^39^. Next, the cells were suspended in RPMI 1640 containing 10% heat-inactivated FBS, 100 units/ml of penicillin, and 100 μg/ml of streptomycin. All cultures were incubated at 37 °C in an atmosphere of 5% CO_2_ in air. FLS were cultured for six to eight passages and lysate was then collected for analyses.

### Statistical analysis

All data were performed using the software GraphPad Prism version 5.0. One-way ANOVA was initially performed to determine whether an overall statistically significant change existed before using the two-tailed paired or unpaired Student’s *t*-test. All values are expressed as the mean ± S.E.M. For each test, *p* values less than 0.05 were considered significant.

## Electronic supplementary material

Supplementary materials for GATA4 regulates angiogenesis and persistence of inflammation in rheumatoid arthritis

## References

[1] Ashraf S, Mapp PI, Walsh DA (2010). Angiogenesis and the persistence of inflammation in a rat model of proliferative synovitis. Arthritis Rheum..

[2] Croft AP (2016). Rheumatoid synovial fibroblasts differentiate into distinct subsets in the presence of cytokines and cartilage. Arthritis Res. Ther..

[3] McInnes IB, Schett G (2011). The pathogenesis of rheumatoid arthritis. N. Engl. J. Med..

[4] Bentley K., Chakravartula S. The temporal basis of angiogenesis. *Philos. Trans. Roy. Soc. B Biol. Sci.* **372**, 1471–2970 (2017). 10.1098/rstb.2015.0522 PMC5379027 28348255

[5] Walsh DA (2010). Angiogenesis and nerve growth factor at the osteochondral junction in rheumatoid arthritis and osteoarthritis. Rheumatology.

[6] Semerano L, Clavel G, Assier E, Denys A, Boissier MC (2011). Blood vessels, a potential therapeutic target in rheumatoid arthritis?. Jt. Bone Spine.

[7] Kong X (2013). Anti-angiogenic effect of triptolide in rheumatoid arthritis by targeting angiogenic cascade. PLoS ONE.

[8] Thairu N, Kiriakidis S, Dawson P, Paleolog E (2011). Angiogenesis as a therapeutic target in arthritis in 2011: learning the lessons of the colorectal cancer experience. Angiogenesis.

[9] Maracle CX, Tas SW (2014). Inhibitors of angiogenesis: ready for prime time?. Best. Pract. Res. Clin. Rheumatol..

[10] Nataraj NB, Krishnamurthy J, Salimath BP (2013). Treatment with anti-NAP monoclonal antibody reduces disease severity in murine model of novel angiogenic protein-induced or ovalbumin-induced arthritis. Clin. Exp. Immunol..

[11] Jay PY (2007). Impaired mesenchymal cell function in Gata4 mutant mice leads to diaphragmatic hernias and primary lung defects. Dev. Biol..

[12] Molkentin JD (2000). The zinc finger-containing transcription factors GATA-4, -5, and -6. Ubiquitously expressed regulators of tissue-specific gene expression. J. Biol. Chem..

[13] Zaytouni T, Efimenko EE, Tevosian SG (2011). GATA transcription factors in the developing reproductive system. Adv. Genet..

[14] Oka T, Xu J, Molkentin JD (2007). Re-employment of developmental transcription factors in adult heart disease. Semin. Cell. Dev. Biol..

[15] Paleolog EM (2009). The vasculature in rheumatoid arthritis: cause or consequence?. Int. J. Exp. Pathol..

[16] Kohli S, Ahuja S, Rani V (2011). Transcription factors in heart: promising therapeutic targets in cardiac hypertrophy. Curr. Cardiol. Rev..

[17] Heineke J (2007). Cardiomyocyte GATA4 functions as a stress-responsive regulator of angiogenesis in the murine heart. J. Clin. Invest..

[18] Heineke J (2008). Cardiomyocyte GATA4 functions as a stress-responsive regulator of angiogenesis in the murine heart (vol 117, pg 3198, 2007). J. Clin. Investig..

[19] Miyazawa K, Mori A, Okudaira H (1998). Establishment and characterization of a novel human rheumatoid fibroblast-like synoviocyte line, MH7A, immortalized with SV40 T antigen. J. Biochem..

[20] Su CM, Huang CY, Tang CH (2016). Characteristics of resistin in rheumatoid arthritis angiogenesis. Biomark. Med..

[21] Maruotti N, Cantatore FP, Ribatti D (2014). Putative effects of potentially anti-angiogenic drugs in rheumatic diseases. Eur. J. Clin. Pharmacol..

[22] Yu W (2016). GATA4 regulates Fgf16 to promote heart repair after injury. Development.

[23] Pakozdi A, Besenyei T, Paragh G, Koch AE, Szekanecz Z (2009). Endothelial progenitor cells in arthritis-associated vasculogenesis and atherosclerosis. Jt. Bone Spine.

[24] Amezcua-Guerra LM, Marquez-Velasco R, Hernandez-Avalos R, Vargas A, Bojalil R (2008). Interferon-gamma is associated with vascular endothelial dysfunction in patients with rheumatoid arthritis. J. Rheumatol..

[25] Wang Z (2014). Berberine ameliorates collagen-induced arthritis in rats associated with anti-inflammatory and anti-angiogenic effects. Inflammation.

[26] Larsen H, Muz B, Khong TL, Feldmann M, Paleolog EM (2012). Differential effects of Th1 versus Th2 cytokines in combination with hypoxia on HIFs and angiogenesis in RA. Arthritis Res. Ther..

[27] Siebert S, Tsoukas A, Robertson J, McInnes I (2015). Cytokines as therapeutic targets in rheumatoid arthritis and other inflammatory diseases. Pharmacol. Rev..

[28] Westra J (2011). Angiopoietin-2 is highly correlated with inflammation and disease activity in recent-onset rheumatoid arthritis and could be predictive for cardiovascular disease. Rheumatology.

[29] Lainer-Carr D, Brahn E (2007). Angiogenesis inhibition as a therapeutic approach for inflammatory synovitis. Nat. Clin. Pract. Rheum..

[30] Chen H (2017). Genome-wide identification, evolution, and expression analysis of GATA transcription factors in apple (Malusxdomestica Borkh.). Gene.

[31] Mazaud Guittot S (2007). The proximal Gata4 promoter directs reporter gene expression to sertoli cells during mouse gonadal development. Biol. Reprod..

[32] Molkentin JD (1998). A calcineurin-dependent transcriptional pathway for cardiac hypertrophy. Cell.

[33] Viger RS, Guittot SM, Anttonen M, Wilson DB, Heikinheimo M (2008). Role of the GATA family of transcription factors in endocrine development, function, and disease. Mol. Endocrinol..

[34] Meek PM, Sood A, Petersen H, Belinsky SA, Tesfaigzi Y (2015). Epigenetic change (GATA-4 gene methylation) is associated with health status in chronic obstructive pulmonary disease. Biol. Res. Nurs..

[35] del Rey MJ (2009). Human inflammatory synovial fibroblasts induce enhanced myeloid cell recruitment and angiogenesis through a hypoxia-inducible transcription factor 1 alpha/vascular endothelial growth factor-mediated pathway in immunodeficient mice. Arthritis Rheum..

[36] Hamilton JL (2016). Targeting VEGF and its receptors for the treatment of osteoarthritis and associated pain. J. Bone Miner. Res..

[37] Koch AE, Distler O (2007). Vasculopathy and disordered angiogenesis in selected rheumatic diseases: rheumatoid arthritis and systemic sclerosis. Arthritis Res. Ther..

[38] Lindblad SS (2009). Smoking and nicotine exposure delay development of collagen-induced arthritis in mice. Arthritis Res. Ther..

[39] Kawahito Y (2000). 15-deoxy-delta(12,14)-PGJ(2) induces synoviocyte apoptosis and suppresses adjuvant-induced arthritis in rats. J. Clin. Invest..

