# Supplementary material for: GATA4 regulates angiogenesis and persistence of inflammation in rheumatoid arthritis
Source: Cell Death Dis. 2018 May 2;9(5):503. doi: 10.1038/s41419-018-0570-5 (PMC5931571; doi:10.1038/s41419-018-0570-5)
Supplement: Supplementary file 1 — Supplementary materials for GATA4 regulates angiogenesis and persistence of inflammation in rheumatoid arthritis [file 41419_2018_570_MOESM1_ESM.docx]

**Supplementary materials for GATA4 regulates angiogenesis and persistence of inflammation in rheumatoid arthritis**

Wanwan Jia ^1,2^, Weijun Wu^1^, Di Yang ^1^, Chenxi Xiao ^1^, Mengwei Huang ^1^, Fen Long ^1^, Zhenghua Su ^1^, Ming Qin ^1^, Xinhua Liu ^1*^, Yi Zhun Zhu ^1,2 *^

**Table S1. Primers used for RT-qPCR validation.**

| Gene name | Primer name | Primer sequence (5´ to 3´) |
| --- | --- | --- |
| bFGF | Human_bFGF_ F | GGGTGCCAGATTAGCGGAC |
|  | Human_bFGF_R | GGTTCACGGATGGGTGTCTC |
| GATA4 | Human_GATA4_F | CAGTCTACGTGCCCACACC |
|  | Human_GATA4_R | TCCCGCCTGGCTCCAT |
| GATA4 | Mouse_GATA4_F | CTGTGCCAACTGCCAGACTA |
|  | Mouse_GATA4_R | TTTGAATCCCCTCCTTCCGC |
| IL-8 | Human_IL-8_F | ACACTGCGCCAACACAGAAA |
|  | Human_IL-8_R | GTTTTCCTTGGGGTCCAGACA |
| VEGF | Human_VEGF_F | ACGGACAGACAGACAGACAC |
|  | Human_VEGF_R | GAAGCGAGAACAGCCCAGAA |
| PEG_2_ | Human _ PEG_2__F | ATGCCAGTAAACAGGCTGACC |
|  | Human _ PEG_2__R | GAAGAAGCCCCCGTTTTTGC |
| GAPDH | Human_GAPDH_F | CTCCAAAATCAAGTGGGGCG |
|  | Human_GAPDH_R | ATGACGAACATGGGGGCATC |
